# Feasibility and Acceptability of Facilitated Advance Care Planning in Outpatient Clinics: A Qualitative Study of Patient and Caregivers Experiences

**DOI:** 10.1177/07334648231206742

**Published:** 2023-11-10

**Authors:** Kate H. Marshall, Diane L. Riddiford-Harland, Anne E. Meller, Gideon A. Caplan, Vasi Naganathan, John Cullen, Peter Gonski, Nicholas A. Zwar, Julie-Ann O’Keeffe, Karolina Krysinska, Joel J. Rhee

**Affiliations:** 1 UNSW Academic General Practice Network, Faculty of Medicine and Health, 7800 University of New South Wales, Sydney, NSW, Australia; 2 School of Medicine, 8691 University of Wollongong, Wollongong, NSW, Australia; 3 Advance Care Planning Services, Prince of Wales Hospital, Sydney, NSW, Australia; 4 Prince of Wales Clinical School, 7800 University of New South Wales, Sydney, NSW, Australia; 5 Department of Geriatric Medicine, Prince of Wales Hospital, Sydney, NSW, Australia; 6 Centre for Education and Research on Ageing (CERA), Department of Geriatric Medicine, Concord Repatriation General Hospital, Sydney, NSW, Australia; 7 Concord Clinical School, Faculty of Medicine and Health, The University of Sydney, Sydney, NSW, Australia; 8 Southcare Aged and Extended Community Care, Sutherland Hospital, Sydney, NSW, Australia; 9 Faculty of Health Sciences and Medicine, 3555 Bond University, Gold Coast, QLD, Australia; 10 Aged, Chronic Care and Rehabilitation, 222415 Sydney Local Health District, Sydney, NSW, Australia; 11 Centre for Primary Health Care and Equity, Faculty of Medicine and Health, 7800 University of New South Wales, Sydney, NSW, Australia; 12 School of Population Health, Faculty of Medicine and Health, 7800 University of New South Wales, Sydney, NSW, Australia

**Keywords:** advance care planning, advanced illness, outpatient care, decision making, end of life

## Abstract

Guidelines recommend advance care planning (ACP) for people with advanced illness; however, evidence supporting ACP as a component of outpatient care is lacking. We sought to establish the feasibility and acceptability of a facilitated ACP intervention for people attending tertiary outpatient clinics. Data from 20 semi-structured interviews with patient (*M* = 79.3 ± 7.7, 60% male) and caregiver (*M* = 68.1 ± 11.0, 60% female) participants recruited as part of a pragmatic, randomized controlled trial (RCT) were analyzed using qualitative descriptive methodology. Patients were randomized to intervention (e.g., facilitated support) or control (e.g., standard care). Intervention patients expressed high satisfaction, reporting the facilitated ACP session was clear, straightforward, and suited to their needs. Intervention caregivers did not report any significant concerns with the facilitated ACP process. Control participants reported greater difficulty completing ACP compared to intervention participants. Embedding facilitated ACP into tertiary outpatient care appears feasible and acceptable for people with advanced illnesses.

**What this paper adds**

• Findings indicate patients with advanced illness treated in outpatient settings and their caregivers perceive facilitated advance care planning (ACP) intervention as feasible and acceptable.
• This study illustrates through qualitative inquiry the key elements of ACP intervention (e.g., facilitated support) which may increase the likelihood of successful implementation of ACP in tertiary outpatient settings.
• Results highlight standard care practices in outpatient services do not consistently lead to ACP for patients with advanced illness.

**Applications of study findings**

• The findings of this study have important implications for the design, development, and delivery of ACP interventions in tertiary outpatient clinics.
• Interventions which include routine screening and assessment, facilitated support and documentation procedures are likely to yield the greatest benefits to patients, their families and health care professionals.
• Ensuring ACP is available and accessible to all patients with advanced illness prior to the onset of critical illness is imperative.

## Background

Advance care planning (ACP) is a process of reflection, discussion, and communication that helps individuals prepare for future medical care at a time when they can no longer communicate decisions for themselves (Sudore et al., 2017). While ACP is widely recognized and endorsed by various professional groups and patient advocates across a range of clinical populations (Houben et al., 2014), considerable individual, service, and system-level challenges (Malhotra & Ramakrishnan, 2022; Risk et al., 2019) have contributed to low uptake across developed countries (Harrison et al., 2016; Knight et al., 2020; White et al., 2014). Integrating ACP interventions into tertiary outpatient clinics, such as geriatric, renal, gastroenterology, hepatology, and respiratory clinics, where individuals receive ongoing speciality care, is one potential strategy to enhance engagement with ACP.

Outpatient clinics offer a unique opportunity for individuals and their families to participate in end-of-life decision-making and planning with healthcare professionals (HCPs). Outpatient clinics may enable individuals with chronic or advanced illnesses to engage in ACP while they are relatively healthy, as well as discuss ACP in the context of their own illness and prognosis. Yet, despite patients’ willingness to engage in ACP, specialist HCPs may miss opportunities to discuss ACP during routine care (Ahluwalia et al., 2012). Lack of clinical time and dedicated space, as well as limited professional education, training, and support have been shown to contribute to poor integration of ACP into outpatient care (Lund et al., 2015).

Tailoring ACP interventions to clinical settings may increase the chances of effective implementation (Lund et al., 2015). Evidence suggests successful ACP interventions typically involve trained HCPs or facilitators, regular screening, and assessment to determine the suitability of ACP, supported ACP discussions, and the chance to complete ACP documentation (e.g., advance directive/plan; De Vleminck et al., 2016). Few studies have assessed ACP implementation in tertiary outpatient settings. Two studies, assessing one multicomponent intervention, found the intervention to be feasible and acceptable from the perspective of HCPs (van Lummel et al., 2022, 2023). Patient and family perceptions of ACP intervention and the extent to which this differs from current care provided in outpatient settings remains unclear. More research is needed to strengthen the evidence-base and inform the implementation of ACP into routine outpatient care.

The present study reports the qualitative results from a multisite, pragmatic randomized controlled trial (RCT) examining the efficacy of a facilitated ACP intervention for people with advanced illness treated in tertiary outpatient clinics (Rhee et al., 2019). As part of the RCT, a subsample of patients and caregivers were interviewed about their experience of ACP intervention and standard care. The objective of this analysis is to explore the feasibility and acceptability of a facilitated ACP intervention from the perspective of patient and caregiver participants, as well as compare the experience of participants who received the intervention and those who received standard care.

## Methods

### Study Design and Sample

Participants in the present study were recruited as part of The Advance Care Planning for Patients with Advanced Illnesses Attending Hospital Outpatient Clinics study, a multisite, pragmatic randomized controlled trial (RCT) of a facilitated ACP intervention for people with advanced illness treated in tertiary outpatient clinics (Rhee et al., 2019). Ethics approval was obtained from South Eastern Sydney Local Health District Human Research Ethics Committee (HREC/16/POWH/654). Patients enrolled in the RCT were aged ≥18 years, receiving care at a subspecialty outpatient clinic who were identified as at risk of dying within 6–12 months using the Supportive and Palliative Care Indicators Tool (SPICT) (Highet et al., 2014), and identified as willing to participate in ACP via the ACP screening tool (Cheang et al., 2014). Consent for patients who lack decision-making capacity was provided by their Person Responsible (i.e., substitute decision-maker in New South Wales, Australia). Caregivers of eligible patients were invited to participate. Exclusion criteria included: (1) patients with an Enduring Guardian and advance directive/plan and were (2) inpatients or permanent residents of a residential aged care facility. Participants with limited reading fluency in English were also excluded. Recruitment processes for the RCT are outlined in the published protocol (Rhee et al., 2019).

Patients and caregivers enrolled in the RCT were invited to participate in the present study six-months post-randomization during follow-up data collection. All participants were asked to indicate their interest for an interview and provided with a hard-copy invitation letter and consent form via their follow-up questionnaire. Consent forms were retuned via reply-paid envelope. Consenting participants were then contacted by the research team (KM) to schedule a telephone interview. To minimize participant burden, individuals identified by the research team as critically unwell were not contacted. Participants were recruited from four groups based on trial randomization; participant group (individuals allocated to receive facilitated ACP [F-ACP], and those who received standard care [SC]), and type (patients [PT] or caregivers [CG]) (Supplementary Figure 1). To ensure the breadth of participants’ clinical experiences were examined, a purposive sampling methodology was used, whereby a sample of patients and caregivers ranging in age, gender, and disease severity were interviewed. Charlson Comorbidity Index (CCI) score defined patient’s disease severity, where higher scores indicate greater morbidity and poorer long-term prognosis (Charlson et al., 1987). Demographic information was captured during baseline data collection as part of the RCT.

### Advance Care Planning Intervention

Features of care provided as part of the intervention included HCP education and training and screening tools to identify and assess patients eligible for ACP. Hard-copy information, ACP documentation (e.g., advance directive/plan), and facilitated support to complete ACP were provided to patients and families. Facilitated support was provided by participating HCPs. Protocols for storing and disseminating ACP documentation were also established at each participating site (Rhee et al., 2019). Intervention characteristics are provided in Table 1. The RCT compared the facilitated ACP intervention to standard clinical care patients and caregivers who are provided with hard-copy information and an advance directive/plan to complete unassisted by themselves (Rhee et al., 2019).

**Table 1.** Overview of Facilitated Advance Care Planning Intervention Implemented in Tertiary Outpatient Clinics.

Component	Features, Processes and/or Procedures
Education and training	• Healthcare professionals (HCPs) are provided with education and training through workshops, lunchtime seminars, or unit meetings by nurse-facilitators. • Training includes identifying and assessing eligible patients, conducting advance care planning (ACP) discussions, legal liability and protection offered by advance care directives and plans (advance directive/plan), and capacity determination. • HCPs are provided with ongoing telephone, email support during the intervention period.
Screening and assessment	• Patients at risk of dying in the next 6–12 months are identified using the Supportive Palliative Indicators Care Tool (SPICT) prior to their appointment. Eligible patients are then approached by a nurse-facilitator or trained HCP.^a^ • Eligible patients and their families are assessed for interest in ACP using the ACP screening tool.^b^ The Person Responsible (i.e., substitute decision maker in New South Wales [NSW]) is approached for patients who lack decision-making capacity
Hard-copy information and advance directive/plan documentation	• Patients and caregivers are provided with hard-copy information on ACP, inlcuding: 1. ACP brochure produced by NSW Health^c^ 2. ACP workbook endorsed by NSW Health^d^ 3. Enduring Guardianship NSW booklet produced by the Public Guardian Office of the NSW Government Department of Attorney General and Justice^e^ 4. Statement of Values and Wishes completed on behalf of the patient by their Person Responsible and supplemental information for families.
Facilitated support to complete ACP	• Patients are provided with personalized information on ACP. • ACP discussions with patients, caregivers, family, and relevant HCPs are faciliated by a nurse-facilitator or trained HCP. • Patients recieved assistance completing appropriate ACP documents (e.g., advance directive/plan). • Referral to relevant local legal services for witnessing are provided as required.
Disseminating advance directive/plan documentation	• Facilitators organized and facilitated meetings and/or teleconferences with outpatient clinic staff, GPs, and other HCPs involved in patient’s care to discuss the patient’s care and their wishes. • Faciliatators assisted with dissemination of ACP documents on completion, that included uploading to EMR record system or national eHealth record which forwarded the document to patient’s general practitioner.

ACP, advance care planning; EMR, electronic medical record; HCP, healthcare professional; NSW, New South Wales; SPICT, supportive palliative indicators Care tool.

^a^Highet et al., 2014.

^b^Cheang et al., 2014.

^c^Health and Social Policy, 2017.

^d^New South Wales Ministry of Health, 2016.

^e^Public Guardian Office of the New South Wales Government Department of Attorney General and Justice, 2011.

### Interviews

Semi-structured telephone interviews were conducted from May to August 2018. Questions were developed with input from experts in the fields of ACP, geriatrics, and service implementation to explore participants’ experiences with facilitated ACP intervention and standard care (Supplementary 1). Proctor et al.’s (Proctor et al., 2011) taxonomy of implementation outcomes were used to inform question development related to feasibility and acceptability. Feasibility and acceptability of ACP screening and assessment was explored across all participants; documentation and facilitated support was additionally explored among participants assigned to receive the intervention. Participants who received standard care were asked to describe their experience of ACP following randomization. Prompting questions were used to follow-up participant answers or seek additional information. Interviews lasted 30–60 minutes and were conducted by two investigators (KM, MH), having no prior relationship with participants. Participants were mailed a $50 (AUD) voucher following interview completion. All interviews were audio-recorded and professionally transcribed.

### Data analysis

Coding was informed by qualitative descriptive methodology (Sandelowski, 2000); themes within each section of the interview were identified through inductive thematic analysis and illustrative quotes extracted. Prominent topics were derived and reviewed by two coders (KM, MH) to increase data reliability. Diverse codes were addressed through discussion; emerging themes were refined through an iterative process of discussion with the analysis team (KM, MH, and JR). Thematic saturation was reached when a stable set of themes emerged, and subsequent interviews did not yield new themes. NVivo (QSR International, Version 12) was used to facilitate qualitative analyses. Descriptive statistics were used to calculate proportions and describe characteristics of participants. Descriptive statistics were analyzed using IBM Statistical Package for the Social Sciences (SPSS) 23.0. Anonymized interview transcripts are available from the corresponding author on reasonable request.

## Results

One-hundred and ninety-seven patients and 132 caregivers were recruited during the trial; 52 patients and 44 caregivers were eligible for interview at 6 months. A sample of ten patients (60% male) and ten caregivers (60% female) were interviewed (Table 2). Mean patient age was 79.3 years (*SD* = 7.7; range 66–99 years) and mean caregiver age was 68.1 years (*SD* = 11.0; range 46–85 years); 70% of participants were born in Australia, 85% were retired, 55% were married and most participants (55%) had completed vocational or tertiary education. Patient CCI scores ranged from 0 to 5 (*M =* 3.0, *SD =* 1.7) and caregivers cared for patients with CCI scores ranging from 0 to 6 (*M* = 2.2, *SD* = 2.0). Three caregivers were identified as their family member’s Person Responsible. Seven caregivers were spousal caregivers and three were adult children.

**Table 2.** Summary Characteristics of Participants Presented by Group.

Characteristics	Participants				Total
	Group 1	Group 2	Group 3	Group 4	
	PT, F-ACP	PT, SC	CG, F-ACP	CG, SC	
	*n* = 7	*n* = 3	*n* = 4	*n* = 6	*N* = 20
Sex					
Male	3	3	2	2	10
Female	3		2	4	10
Age stratification					
>50				1	1
50–59				1	1
60–69		1	2	1	4
70–79	2		1	3	7
80 +	4	2	1		7
Country of birth					
Australia	3	3	3	5	14
New Zealand	1				1
United Kingdom	1			1	2
Other	2		1		3
Employment status					
Part- or full-time employment				2	2
Retired or pensioner	6	3	4	4	17
Marital status					
Not married		2		1	3
Married or de facto relationship	2	1	5	3	11
Separated or divorced	3		1		4
Widowed	2				2
Level of education					
Primary school	1		1		2
Some high school		2		1	3
Completed high school	2		1	1	4
Vocational or tertiary education	4	1	2	4	11
Charlson Comorbidity Index score^a^					
0	2			2	4
1–2	1		2	2	5
3–4	4	2	2	1	9
>5	-	1		1	2

PT, patient; CG, caregiver; F-ACP, facilitated advance care planning; SC, standard care (written information only).

^a^Scores calculated for patients only. Higher scores indicate greater morbidity and poorer long-term prognosis.

Results are presented as specific to the perspective of patients who received facilitated ACP (Group 1 [PT, F-ACP]) and patients who received standard care (Group 2 [PT, SC]), and specific to the perspective of caregivers of patients who received facilitated ACP (Group 3 [CG, F-ACP]) and caregivers of patients who received standard care (Group 4 [CG, SC]).

### Advance Care Planning Screening and Assessment

As no observable differences were evident, data from both patient (Groups 1 and 2) and caregiver (Groups 3 and 4) groups were combined. Groups 1 and 2 responded positively to being approached to participate in ACP during their outpatient appointment (10/10, 100%). Patients reported ACP as relevant to their situation, stating it was an appropriate time to start discussing and planning end-of-life care considering their age and illness status (2/10, 20%)*.* Most caregivers (Groups 3 and 4) expressed no significant concerns regarding their family member’s participation in ACP (7/10, 70%); however, some caregivers (3/10, 30%) indicated they were apprehensive to participate. Caregivers described a sense of relief that their family member had been asked to engage in ACP (4/10, 40%*;* “*I was so glad that a third person had suggested it and it’s not something that I had to suggest to my mother”* [Female, 46, CG, SC]).

When asked about their motivations to participate in ACP, patients indicated the process would be personally beneficial (5/10, 50%) and help clarify their end-of-life wishes (5/10, 50%). Patients cited completing ACP would reduce the burden placed on their family if their health was to decline (“*I believe it’s not about me, it’s about my family when I’m in a situation where I can’t do anything. I don’t want my family to suffer”* [Female, 75, PT, F-ACP]). Patients also described a desire to be organized, believing ACP was a normal part of preparing for the future (2/10, 20%). Caregivers anticipated that participating in ACP would help them understand their family members’ end-of-life care preferences (7/10, 70%). Caregivers also considered ACP to be an important step in preparing for the future (“*You’re looking ahead to what might happen in the future and getting yourself prepared - that’s why it’s a good idea”* [Male, 66, CG, F-ACP]).

### Facilitated Advance Care Planning Intervention

Group 1 frequently described a high level of satisfaction with the session provided by the nurse-facilitators (7/7, 100%). Patients reported the facilitated ACP discussion was clear, and the process of completing ACP documentation during the session was straightforward (4/7, 57%). Patients reported feeling comfortable throughout the session (7/7, 100%), with some describing that the nurse-facilitators guidance eased their anxiety (2/7, 29%). As one patient described, *“They were very understanding. For the things I was apprehensive about, they explained and made me feel more comfortable”* [Female, 75, PT, F-ACP]. Many patients perceived the discussion to be open and interactive (4/7, 57%), indicating throughout the session, the nurse-facilitators answered their questions and concerns appropriately (7/7, 100%). Most patients were satisfied with the length of the discussion (5/7, 71%) and described the session as suitable to their information needs (5/7, 71%). All Group 1 participants completed an advance directive during the facilitated session (7/7, 100%).

Most Group 1 patients reported participating in the ACP discussion helped clarify their wishes for their end-of-life care (6/7, 86%; *“It was very helpful because I hadn’t actually thought of the details of how I would like to have my last days”* [Female, 82, PT, F-ACP]). Others were appreciative of the conversation (*“It brought it out in the open how serious all this was and how important it was to finalise it”* [Female, 75, PT, F-ACP]). Most of Group 1 indicated the discussion did not impact the relationship between themselves and their friends and family (4/7, 57%). Participating in ACP did not impact patient’s relationship with their current HCP (6/7, 85%); however, the majority indicated they had not spoken with their HCP about their completed advance directive (4/7, 57%).

All Group 3 caregivers participated in their family member’s facilitated ACP session. All described attending the session and only listening to the patient’s answers. Caregivers felt attending the facilitated ACP session allowed them to better understand their family member’s wishes, (2/4, 50%*)*; *“I think it was good that the two of us were there. I got his thoughts on it and if the time should come, it makes things so much easier to know what that person wanted done or wants done”* [Female, 67, CG, F-ACP]). Caregivers enjoyed the interactive and open nature of the discussion (4/4, 100%) and cited the session was an appropriate length of time (3/4, 75%). Throughout the session, all caregivers felt their questions were addressed by the nurse-facilitators, and no one cited issues with the structure and content of the session. One caregiver described how it was important to have an impartial person assist with care decisions, stating, *“It was somebody that didn’t know you, so they had no interest or ulterior motive to push you or to decide one way or the other. They were impartial, so whatever he wanted was noted down and nobody tried to change his mind or select another option”* [Female, 70, CG, F-ACP].

Group 3 caregivers felt the facilitated ACP session helped clarify their family members’ wishes (3/4, 75%; “*I think it was very beneficial to know that he was quite unsure really of how he would feel, and basically said he would just leave it up to the family at the time to make the decision”* [Female, 67, CG, F-ACP]). Overall, caregivers were positive about the impact of the session on themselves and their family (4/4, 100%). As one caregiver remarked, *“It was strangely a positive one, because it’s a relief to have the hard conversation. By having the document done with Mum and the family, it turned what could have been a negative into a positive”* [Female, 55, CG, F-ACP]). Caregivers did not report any changes to their relationship with the patients’ health care team following the session (4/4, 100%).

### Participant Experience of Standard Care

Among Group 2 patients, none engaged in ACP resulting in an advance directive after they were provided with written information. Common to these patients was an appreciation of the importance of ACP discussions but many were unsure how to complete ACP documentation by themselves (2/3, 67%) or were uncertain about their end-of-life wishes (2/3, 67%). Most Group 2 patients reported that without follow-up from an HCP they felt ACP was no longer relevant to them (2/3, 67%). Although all had spoken briefly with their family and friends about their end-of-life wishes since receiving the information, patients reported these conversations were not formally documented (3/3, 100%). When asked whether they had spoken to a health professional about ACP, only one patient had discussed their end-of-life wishes with their GP. Other patients were not aware they could discuss ACP with their GP (2/3, 67%), with one patient stating their GP would prioritize ACP (*“I could speak with my GP, but I don’t think they would be terribly interested”* [Male, 82, PT, SC]).

Group 4 caregivers reported having multiple roles in the ACP process including, explaining medical and treatment information to their ill family member (3/6, 50%), providing emotional support (2/6, 33%) and coordinating care decisions with extended family members (1/6, 17%). As one caregiver described, *“My role was to organise Mum to be aware of what the documents said and to let her know what ACP is - so what happens if you have a stroke, explain that to her, what could happen, whether you want resuscitation, or you want to not, or you want to go into a nursing home. We had all the very hard conversations. My role was to have those with her and then also bring the family in and coordinate with the GP and the family. My role was basically coordinate Mum’s wishes to everybody else”* [Female, 55, CG, SC]. Caregivers spoke most frequently to family and friends about ACP after their family member had received the ACP documents (6/6, 100%). Most Group 4 caregivers had also attended a GP appointment with their family member where ACP was raised (4/6, 67%). A small proportion of Group 4 caregivers did not participate in ACP with their family member (2/6, 33%); citing they felt either apprehensive to make care decisions on their family member’s behalf (2/6, 33%), or too distressed (1/6, 17%) or time poor to complete ACP (1/6, 17%).

Three Group 4 caregivers were identified as their family member’s Person Responsible; two described completing ACP with their family member. Both caregivers reported significant challenges facilitating the ACP process. Caregivers reported feeling uncomfortable guiding their family member through ACP because they did not have sufficient medical knowledge. As one caregiver noted, *“I felt like I was almost answering the questions for her, and giving her suggestions on what she should do, and she would just go with what I suggested. I felt like I was leading her in some way which made me feel uncomfortable. I think if we had done it with a doctor who could guide us through what the medical options are in those situations, then, we would’ve better understood what to do, because we were just guessing really”* [Female, 46, CG, SC]. Caregivers also found it difficult to seek support from HCPs to complete a Statement of Values and Wishes (*“I was hoping the doctor would go through it with my mum and just have a look at what we’d written, but the doctor didn’t want to sign it because she wasn’t sure about my mum’s capacity”* [Female, 46, CG, SC]). Both caregivers were upset that an advance directive had not been suggested prior to the patient losing capacity, with one caregiver stating, *“I was told that, because he doesn’t have capacity, you can’t do an Advance Care Directive. That was a huge shock and disappointment to me because I’ve had doctors going on and on and on about, ‘Oh, you’ve got to do an Advance Care Directive’. Nobody told me, now he doesn’t have capacity and he can’t do one. I felt really left out”* [Female, 69, CG, SC].

## Discussion

This study explored the feasibility and acceptability of a facilitated ACP intervention for people with advanced illness treated in tertiary outpatient clinics. All patients reported they were comfortable being approached to participate in ACP during their outpatient appointment. Several elements of the intervention contributed to participant’s satisfaction with care. Most notably, patients and caregivers who received the intervention described a high level of satisfaction with the timing, content, and delivery of the facilitated ACP session. Intervention patients believed the facilitators were attentive to their information and supportive care needs; caregivers of patients who received facilitated ACP were also appreciative of the facilitators approach to the session.

Unique to this study was the ability to compare the experience of participants who received facilitated support and standard care. Patients provided with facilitated support completed ACP documents such as advance directives; however, none of the patients who received standard care (i.e., hard-copy information and ACP documentation only) reported completing ACP documents. Evidence suggests individual barriers such as perceiving ACP as irrelevant, poor understanding of ACP processes and documentation, and emotional difficulties (e.g., uncertainty, distress), can adversely affect all stages of the ACP process (Freer et al., 2006; Morrison & Meier, 2004; Schickedanz et al., 2009). While many patients expect HCPs to initiate and support ACP conversations (Bernard et al., 2020; De Vleminck et al., 2013), our findings indicate these expectations may remain even after patients have received standard care, resulting in incomplete ACP documentation. Engaging with facilitators throughout the ACP process may help overcome individual-level challenges and improve patient engagement with ACP and uptake of documentation.

Caregivers of patients provided with standard care reported taking responsibility for facilitating the ACP process. Being approached to participate in ACP may have spurred an increase in caregivers' willingness to engage in ACP conversations; while providing written information may have motivated caregivers to assist their family members in completing ACP documentation. Studies indicate caregivers often assist family members in ACP discussions by clarifying potential medical outcomes and treatment options (Dionne-Odom et al., 2019; Lamore et al., 2017), and collaborating with HCPs to make care decisions (Dionne-Odom et al., 2019; Laidsaar-Powell et al., 2013). Yet, these forms of supportive roles could potentially impact decision-making, causing patients to make decisions that conform to their caregivers' values rather than their own (Dionne-Odom et al., 2019). This poses a significant challenge as patient and caregiver end-of-life preferences do not always align (Shin et al., 2015; Tyrrell et al., 2006). Nurse-led ACP facilitation has been found to increase congruence between patients and their caregivers understanding of their end-of-life treatment and care preferences compared to standard care practices (i.e., written information) (Schwartz et al., 2002). Facilitated ACP interventions may help to minimize disparities between patients' preferences and the care they receive at the end of their lives.

Caregivers (two of three) who identified as their family member’s Person Responsible (legal surrogate decision-maker) expressed feeling hesitant about making care decisions on the patient’s behalf after receiving standard care. Both caregivers reported feeling uneasy about leading the conversation, as they believed they lacked the necessary medical expertise to adequately assist their family member. Caregivers of people with impaired capacity experience considerable challenges as surrogate decision-makers (Heyland et al., 2003; Hines et al., 2001). Caregivers report unmet informational needs regarding the patient’s prognosis, future treatment options, and management of end-of-life symptoms (Hwang et al., 2003; Mangan Jr. et al., 2003; Wang et al., 2018; Washington et al., 2011), which can impede their ability to accurately anticipate a patient’s end-of-life preferences (Jeong et al., 2011; Uhlmann et al., 1988). Moreover, caregivers reported difficulties seeking support from HCPs to complete ACP when provided with only written information. Given the likelihood that these caregivers may experience feelings of isolation and high levels of psychological distress (Brodaty & Donkin, 2009), these findings underscore the necessity for tailored ACP support for caregivers of individuals with limited capacity.

### Strengths and Limitations

Qualitative methodology plays a valuable role in exploring patient experiences and is widely acknowledged as best practice for evaluating complex health interventions (Datta & Petticrew, 2013). Qualitative data in the field of ACP implementation can be used to complement quantitative finding, and this is particularly relevant given the increasing criticism regarding the efficacy of ACP (Jimenez et al., 2018; Morrison et al., 2021). Including perspectives from both patients and caregivers, as well as the comparison of participants who received facilitated ACP intervention and standard care, are also key strengths of this study.

Nevertheless, several factors must be considered when interpreting our findings. Recruitment for this study occurred six-months after individuals were randomized as part of the RCT and this may have limited our ability to recruit patients with higher medical acuity. Our sample included predominantly older adults (aged ≥50 years) despite patients aged ≥18 years being eligible to participate, reflecting the older population accessing outpatient clinics. As data was not collected on nonrespondents, it is possible those who did not participate in the present study had a different experience. Only 4% of patients assigned to the standard care group completed an advance directive/plan, and due to this small number, we were unable to secure an interview with a participant from this subsample. Participants were recruited from metropolitan-based outpatient clinics in Sydney, Australia, limiting the generalizability of our results to regional and rural areas, and other healthcare systems. Most participants were Australian-born, English-speaking, and tertiary educated, influencing the diversity of our sample. Interviews required access to a telephone, and this may have excluded vulnerable populations. It is possible caregivers with more intensive caring responsibilities may not have been able to complete an interview due to limited time. Participants reported more positives about the intervention than negatives, suggesting social desirability (Bergen & Labonté, 2019) may have prompted positive rather than negative answers. Not all participants commented on identified themes during the interview, thus impacting our ability to accurately determine the proportion of individuals who held a particular view. Data was also captured prior to the coronavirus disease (COVID-19) pandemic. It is possible COVID-19 may impact ACP implementation in outpatient settings.

## Conclusions

Facilitated ACP intervention which supports patients and caregivers through multiple elements of the ACP process appears both feasible and acceptable in outpatient settings. Our findings highlight current care practices implemented in tertiary outpatient clinics do not consistently lead to ACP. Active participation and communication with facilitators throughout the ACP process could enhance engagement among patients and caregivers by addressing typical individual, service, and system-related obstacles to ACP adoption. Strengths and limitations identified by patients and caregivers will assist in intervention refinement and complement future efficacy data. Further research is required to establish the effectiveness of ACP intervention and enhance our understanding of the factors that impact ACP implementation and documentation in tertiary outpatient settings.

## Supplemental Material

Supplemental Material - Feasibility and Acceptability of Facilitated Advance Care Planning in Outpatient Clinics: A Qualitative Study of Patient and Caregivers Experiences Click here for additional data file. Supplemental Material for Feasibility and Acceptability of Facilitated Advance Care Planning in Outpatient Clinics: A Qualitative Study of Patient and Caregivers Experiences by Kate H. Marshall, Diane L. Riddiford-Harland, Anne E. Meller, Gideon A. Caplan, Vasi Naganathan, John Cullen, Peter Gonski, Nicholas Zwar, Julie-Ann O’Keeffe, Karolina Krysinska, and Joel J. Rhee in Journal of Applied Gerontology
