# Supplementary material for: Feasibility and Acceptability of Facilitated Advance Care Planning in Outpatient Clinics: A Qualitative Study of Patient and Caregivers Experiences
Source: J Appl Gerontol. 2023 Nov 10;43(4):339–48. doi: 10.1177/07334648231206742 (PMC10875901; doi:10.1177/07334648231206742)
Supplement: Supplemental Material - Feasibility and Acceptability of Facilitated Advance Care Planning in Outpatient Clinics: A Qualitative Study of Patient and Caregivers Experiences [file sj-pdf-1-jag-10.1177_07334648231206742.pdf]

Supplementary Material

Supplementary Table 1. Semi-structured interview schedule

Topic of discussion	Group*	Schedule^
Being approached to participate in ACP	1, 2	• How did you feel when you were approached to participate in the study? *Did you feel that ACP was relevant to you and your family? Did being approached raise any concerns for you? Did you feel the timing was right?*<br>• What was your motivation to participate in the study?
	3, 4	• How did you feel when your family member/friend was approached to participate in the study? *Did you feel that ACP was relevant to you and your family member? Did being approached raise any concerns for you?*
Experience of ACP	1	• Tell me about the session that you had on Advance Care Planning. *How did it start? Who facilitated it? Who else was present? What were your initial expectations? How did you feel about the discussion? Did you feel comfortable?*<br>• How open and interactive did you feel the sessions were?<br>• How did you find the length of the discussion?<br>• Were your questions and concerns adequately addressed during the discussion?
	2	• What did you do after you were given the ACP documents?<br>• Did you speak with anyone about ACP? Did the discussion raise any concerns for you?<br>• Did you need/seek assistance to complete the ACP documents?<br>• Did you speak with your family members/friends/caregiver about your wishes? *Why/why not? Were you comfortable talking to your family members about ACP?*<br>• Did you speak with your GP about your wishes? *Why/why not? Were you comfortable speaking to your GP about ACP?*<br>• Did you speak with specialists or hospital staff about your AC wishes? *Why/why not? Were you comfortable speaking to staff about ACP?*
	3	• Tell me about the session that you and your family member had about Advance Care Planning. *How did it start? Who else was there? What happened? What role did you play? What were your initial expectations? How did you feel about the discussion? Did you feel comfortable?*<br>• How open and interactive did you feel the session was?<br>• How did you find the length of the session?<br>• Were your question and concerns adequately addressed during the session?
	4	• What was your role after [*patient name*] was given the ACP documents? *Were you appointed as an Enduring Guardian, identified as person responsible etc.?*<br>• Were you part of any ACP discussions with [*patient name*]? *Did the discussion raise any concerns for you?*<br>• Did you need or seek assistance to complete the ACP documents?<br>• Did you speak with [*patient name*] and other family members/friends about [*patient name*]'s wishes? *Why/why not? Were you comfortable talking to your family members about ACP?*
Impact of facilitated ACP	1	• How did the ACP session affect your thoughts, wishes and priorities about your future care?<br>• Did the session affect your ability to communicate your wishes with your family and/or friends?<br>• Did the session affect your ability to communicate your wishes with your health care team?

Topic of discussion	Group*	Schedule^
		• How did the session affect the relationship between you and your family or friends?<br>• How did the session affect the relationship between you and your health care team?
	3	• How did the discussion affect your understanding of [*patient name*]'s thoughts, wishes and priorities for the future?<br>• How did the discussion affect your relationship with [*patient name*] or other family members/friends?<br>• How did the discussion affect the relationship between you and [*patient name*]'s health care team?
Barriers to ACP	1	• Were there any issues or problems that you encountered during the session?
	2	• Can you tell me about any problems or barriers that you encountered when discussing ACP and/or completing the ACD?
	3	• Were there any issues or problems that you encountered during the session?
	4	• Can you tell me about any problems or barriers that you encountered when discussing ACP and/or completing the ACD?

ACP, Advance Care Planning

*Group 1= patient, facilitated advance care planning (F-ACP); Group 2= patient, standard care (SC); Group 3= caregiver, F-ACP; Group 4= caregiver, SC

^Questions in italics were used by the interviewer as prompts when required in order to explore the participant's response.

Supplementary Table 2. Consolidated criteria for reporting qualitative studies (COREQ), 32-item checklist.

Developed from: Tong, A., Sainsbury, P., & Craig, J. (2007). Consolidated criteria for reporting qualitative research (COREQ): a 32-item checklist for interviews and focus groups. Int J Qual Health Care, 19(6), 349-357. https://doi.org/10.1093/intqhc/mzm042

Item	Description	Reported on page number
**Domain 1: Research team and reflexivity**		
Personal characteristics		
1. Interviewer/ facilitator<br>Which author/s conducted the interview or focus group?	Interviews were conducted by Ms. Kate Marshall and Dr Mohammed Hossain.	Methods, page 4
2. Credentials<br>What were the researcher's credentials? E.g. PhD, MD	**Research team:**<br>Kate H. Marshall B Psych (Hons), Diane L. Riddiford-Harland PhD, Anne E. Meller BSc, Gideon A. Caplan MBBS, MD, FRACP, Vasi Naganathan MBBS, PhD, FRACP, John Cullen MBBS(Hons), FRACP, Peter Gonski MBBS, FRACP, Nicholas Zwar MBBS, PhD, FRACGP, Julie-Ann O'Keeffe BSc, Karolina Krysinska PhD, Joel J. Rhee MBBS, PhD, FRACGP, Mohammed Hossain, PhD	Title page

3. Occupation<br>What was their occupation at the time of the study?	Kate H. Marshall: Research Officer<br>Diane L. Riddiford-Harland: Research Fellow<br>Anne E. Meller; Clinical nurse consultant<br>Gideon A. Caplan: Consultant, Professor<br>Vasi Naganathan: Consultant, Professor<br>John Cullen: Consultant<br>Peter Gonski: Consultant, Associate Professor<br>Nicholas Zwar: Professor<br>Julie-Ann O'Keeffe: Operations manager<br>Karolina Krysinska: Research Fellow<br>Joel J. Rhee: Consultant, Associate Professor<br>Mohammad Hossain: Research Officer	Title page
4. Gender Female – Was the researcher male or female?	6 male (54%), 5 female (45%)	-
5. Experience and training<br>What experience or training did the researcher have?	All researchers had prior experience in qualitative health research.	Methods, page 6-7
Relationship with participants		
6. Relationship established<br>Was a relationship established prior to study commencement?	Participants in the present study were recruited as part of The Advance Care Planning for Patients with Advanced Illnesses Attending Hospital Outpatient Clinics study, a multisite, pragmatic randomized controlled trial (RCT) of a facilitated ACP intervention for people with advanced illness treated in outpatient clinics (Rhee et al., 2019)<br><br>Recruitment for the RCT was completed by clinical nurse consultants. Researchers did not have a prior relationship with the interviewers.<br><br>Reference<br>Rhee, J., Meller, A., Krysinska, K., Gonski, P., Naganathan, V., Zwar, N., Hayen, A., Cullen, J., O'Keefe, J. A., McDonald, J., Harris-Roxas, B., & Caplan, G. A. (2019). Advance care planning for patients with advanced illnesses attending hospital outpatient clinics study: a study protocol for a randomised controlled trial. BMJ Open, 9(1), e023107. https://doi.org/10.1136/bmjopen-2018-023107	-
7. Participant knowledge of the interviewer<br>What did the participants know about the researcher? e.g. personal goals, reasons for doing the research	Participants were informed of the study aims using hard-copy participant information sheets and consent forms.	Methods, page 5-6
8. Interviewer characteristics<br>What characteristics were reported about the interviewer/facilitator? e.g. Bias, assumptions, reasons	Interviewers were skilled qualitative researchers with prior experience in health research examining chronically ill populations.	-

and interests in the research topic		
**Domain 2: Study design**		
Theoretical framework		
9. Methodological orientation and Theory<br>What methodological orientation was stated to underpin the study? e.g. grounded theory, discourse analysis, ethnography, phenomenology, content analysis	Coding was informed by qualitative descriptive methodology (Sandelowski, 2000).<br><br>Sandelowski, M. (2000). Whatever happened to qualitative description? *Research in nursing & health*, *23*(4), 334-340.	Methods, page 10.
Participant selection		
10. Sampling<br>How were participants selected? e.g. purposive, convenience, consecutive, snowball	A purposive sampling methodology was used, whereby a sample of patients and caregivers ranging in age, gender, and disease severity were interviewed.	Methods, page 7
11. Method of approach<br>How were participants approached? e.g. face-to-face, telephone, mail, email	Participants in the present study were recruited as part of The Advance Care Planning for Patients with Advanced Illnesses Attending Hospital Outpatient Clinics study, a multisite, pragmatic randomized controlled trial (RCT) of a facilitated ACP intervention for people with advanced illness treated in outpatient clinics (Rhee et al., 2019).<br><br>Patients and caregivers enrolled in the RCT were invited to participate in the present study six-months post-randomisation during follow-up data collection. All participants were asked to indicate their interest for an interview and provided with a hard-copy invitation letter and consent form via their follow-up questionnaire. Consent forms were retuned via reply-paid envelope. Consenting participants were then contacted by the research team (KM) to schedule a telephone interview.	Methods, page 7
12. Sample size<br>How many participants were in the study?	20 participants, including 10 patients and 10 caregivers.	Results, page 7
13. Non-participation<br>How many people refused to participate or dropped out? Reasons?	One-hundred and ninety-seven patients and 132 caregivers were recruited during the trial; 52 patients and 44 caregivers were eligible for interview at six-months. Participants indicated interest during 6-month data collection. Ten patients (19% of eligible, contactable adults) and 10 caregivers (22%) were recruited.	Results, page 7
Setting		
14. Setting of data collection<br>Where was the data collected? e.g. home, clinic, workplace	All interviews were conducted via telephone. Researchers conducted calls from the University of New South Wales.	Methods, page 4-5

15. Presence of non-participants Was anyone else present besides the participants and researchers?	It is possible that family members were present during the phone interviews.	-
16. Description of sample What are the important characteristics of the sample? e.g. demographic data, date	A sample of ten patients (60% male) and ten caregivers (60% female) were interviewed. Mean patient age was 79.3 years (SD=7.7; range 66-99 years) and mean caregiver age was 68.1 years (SD=11; range 46-85 years); 70% of participants were born in Australia, 85% were retired, 55% were married and most participants (55%) had completed vocational or tertiary education. Patient CCI scores ranged from 0 to 5 (M=3.0, SD=1.7) and caregivers cared for patients with CCI scores ranging from 0 to 6 (M=2.2, SD=2.0). Three caregivers were identified as their family member's Person Responsible. Seven caregivers were spousal caregivers and three were adult children.	Results, page 7-8
Data collection		
17. Interview guide Were questions, prompts, guides provided by the authors? Was it pilot tested?	Outlined in Supplementary Table 1.	Methods, page 4-5. Sample interview questions provided in Supplementary. Interview guides were not pilot tested.
18. Repeat interviews Were repeat interviews carried out? If yes, how many?	Not applicable.	-
19. Audio/visual recording Did the research use audio or visual recording to collect the data?	Yes. Interviews were audio recorded.	Methods, page 6
20. Field notes Were field notes made during and/or after the interview or focus group?	No field notes were not captured.	-
21. Duration What was the duration of the interviews or focus group?	30-60 minutes	Methods, page 6
22. Data saturation Was data saturation discussed?	Thematic saturation was reached when a stable set of themes emerged, and subsequent interviews did not yield new themes.	Methods, page 7
23. Transcripts returned Were transcripts returned to participants for comment and/or correction?	Participants were not sent transcripts for comment.	-
**Domain 3: Analysis and findings**		

Data analysis		
24. Number of data coders How many data coders coded the data?	Prominent topics were derived and reviewed by two coders (KM, MH) to increase data reliability.	Methods, page 7
25. Description of the coding tree Did authors provide a description of the coding tree?	Not applicable.	-
26. Derivation of themes Were themes identified in advance or derived from the data?	Diverse codes were addressed through discussion; emerging themes were refined through an iterative process of discussion with the analysis team (KM, MH, and JR).	Methods, page 7
27. Software What software, if applicable, was used to manage the data?	NVivo (QSR International, Version 12) was used to facilitate qualitative analyses.	Methods, page 7
28. Participant checking Did participants provide feedback on the findings?	Participants did not provide feedback.	-
Reporting		
29. Quotations presented Were participant quotations presented to illustrate the themes / findings? Was each quotation identified? e.g. participant number	Results are presented as specific to the perspective of patients who received facilitated ACP (Group 1; PT, F-ACP) and patients who received standard care (Group 2; PT, SC), and specific to the perspective of caregivers of patients who received facilitated ACP (Group 3; CG, F-ACP) and caregivers of patients who received standard care (Group 4; CG, SC).	Results, page 7-8
30. Data and findings consistent Was there consistency between the data presented and the findings?	Yes, the participant quotations follow the NVivo coding structure.	-
31. Clarity of major themes Were major themes clearly presented in the findings?	For this study, data was presented on the feasibility and acceptability of the elements of ACP intervention. Results are presented on two key elements: (1) ACP screening and assessment and (2) facilitated ACP intervention. The experiences of people who were given the intervention are analysed and compared with those who received the usual or standard care; these data are presented in the Results.	Results, page 7-13
32. Clarity of minor themes Is there a description of diverse cases or discussion of minor themes?	Minor themes are explored in the Results.	Results, page 7-13

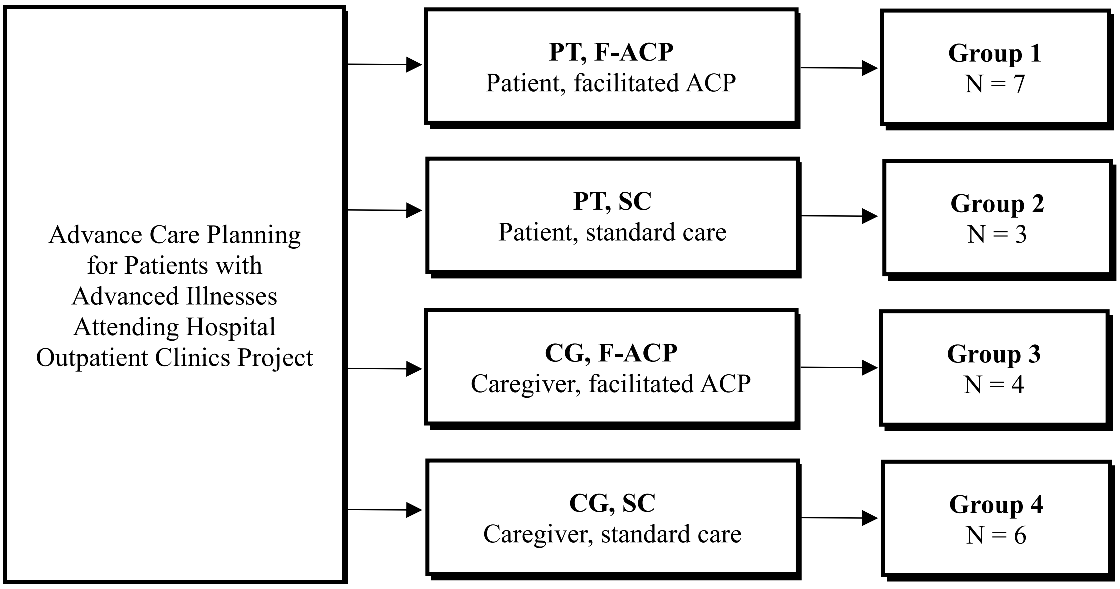

Supplementary Figure 1. Participant recruitment strategy
